# A novel prognostic model for predicting the risk of first variceal hemorrhage in patients with HBV-related cirrhosis

**DOI:** 10.3389/fcimb.2023.1062172

**Published:** 2023-01-17

**Authors:** Qun Zhang, Shuaishuai Niu, Li Yang, Bingbing Zhu, Ke Shi, Xiaohua Zhang, Yi Zhang, Yufei Bi, Yongping Mu, Xianbo Wang

**Affiliations:** ^1^ Center of Integrative Medicine, Beijing Ditan Hospital, Capital Medical University, Beijing, China; ^2^ Institute of Liver Diseases, Shuguang Hospital Affiliated to Shanghai University of Traditional Chinese Medicine, Shanghai, China

**Keywords:** gastroesophageal varices, hepatitis B virus, liver cirrhosi, nomogram, variceal hemorrhage, prognostic model

## Abstract

**Background:**

Variceal hemorrhage (VH) is a life-threatening complication of cirrhosis. An accurate VH risk evaluation is critical to determine appropriate prevention strategies. We aimed to develop an individualized prediction model to predict the risk of first VH in hepatitis B virus (HBV)-related cirrhotic patients.

**Methods:**

A nomogram was developed based on a retrospective analysis of 527 consecutive HBV-related cirrhotic patients with gastroesophageal varices (GEVs). The nomogram evaluation was performed using the area under the receiver operating characteristic curve (AUC), concordance index (C-index), calibration plot, and decision curve analysis (DCA). The results were verified using an external cohort (n = 187).

**Results:**

We developed a nomogram based on clinical and endoscopic features, including the size of varices, red wale marks, ascites, spleen thickness, γ‐glutamyltransferase, and hematocrit. The C-index of the nomogram in the derivation and validation cohort was 0.806 and 0.820, respectively, and the calibration plot fitted well. Compared with those of the North Italian Endoscopic Club (NIEC) and revised NIEC indexes, the AUC (derivation cohort: 0.822 vs. 0.653 vs. 0.713; validation cohort: 0.846 vs. 0.685 vs. 0.747) and DCA curves of this nomogram were better. Further, based on the risk scores, patients were classified into low-, medium-, and high-risk groups, and significant differences were noted in VH incidence among the three risk groups (*P <*0.001 for each cohort).

**Conclusions:**

An effective individualized nomogram to predict the risk of first VH in HBV-related GEV patients was established, which can assist clinicians in developing more appropriate prevention strategies.

## 1 Introduction

Variceal hemorrhage (VH) is a serious complication of cirrhotic portal hypertension and one of the leading causes of death worldwide (La Mura et al., 2020). The annual incidence of first bleeding events in patients with gastroesophageal varices (GEVs) is 5–15%, and more than 15% of the initial bleeding episodes are fatal (Shukla et al., 2016; Haq and Tripathi, 2017). Despite progress in diagnosis and therapy of VH, the mortality from the initial bleeding episode remains high (Reverter et al., 2014). Even if the bleeding is controlled, the patients still have very high-risk of recurrent bleeding, which is associated with mortality as high as that of the first bleed (O’Brien et al., 2013). Hepatitis B virus (HBV) infection is a serious global public health problem (Revill et al., 2020), and a large proportion of GEV cases is associated with HBV infection (Liu et al., 2016; Lv et al., 2019). Reducing the incidence of HBV-related VH is important to decrease the overall mortality rate associated with cirrhosis. For this purpose, routine bleeding risk assessment in patients with HBV-related GEVs is essential such that appropriate prophylactic measures are administered to avoid the first VH episode occurrence.

Hepatic venous pressure gradient (HVPG) is considered an excellent VH predictor (La Mura et al., 2020). Patients with HVPG ≥12 mmHg face high VH risk; in contrast, this bleeding risk significantly decreases when HVPG is <12 mmHg or when the HVPG is reduced by more than 20% from the baseline level (Eisenbrey et al., 2013; La Mura et al., 2020). However, the HVPG measurement is an invasive procedure, which is not suitable for routine clinical testing in most patients. Generally, the stratification strategy for GEVs is mainly based on endoscopic screening, which is currently recommended by the guidelines for cirrhotic patients (Garcia-Tsao et al., 2017). The size of varices >5 mm and appearance of red wale marks (RWM) are considered as high-risk features of VH (The North Italian Endoscopic Club for the Study and Treatment of Esophageal Varices, 1988). However, a study reported that only 30% of patients with actual bleeding presented with these endoscopic risk features (The North Italian Endoscopic Club for the Study and Treatment of Esophageal Varices, 1988). Therefore, endoscopic screening alone may not be enough to accurately identify patients with high-risk of VH occurrence.

The combination of the clinical indicators and endoscopic features is considered as the appropriate tool to provide a better assessment of VH risk (The North Italian Endoscopic Club for the Study and Treatment of Esophageal Varices, 1988). Currently, the most widely used indexes to stratify high-risk patients are the North Italian Endoscopic Club described NIEC index and revised (Rev)-NIEC index that was proposed by Carlo Merkel et al. (The North Italian Endoscopic Club for the Study and Treatment of Esophageal Varices, 1988; Merkel et al., 2000). Both indexes are a combination of the Child-Pugh classification and endoscopic parameters including the size of varices and RWM (The North Italian Endoscopic Club for the Study and Treatment of Esophageal Varices, 1988; Merkel et al., 2000). However, both the NIEC and Rev-NIEC indexes were developed primarily based on the data on populations with alcohol issues and/or hepatitis C virus infections. Whether they can accurately predict the VH risk of HBV-related GEV patients is unknown. In addition, these models were proposed more than two decades ago. Considerable advances have been made in liver disease treatment in the past 20 years, especially toward the antiviral treatment of HBV infection (Wang and Duan, 2021). As the medical environment changes, the predictive performance of these models may also change. Moreover, despite the greatly improved outcomes of antiviral therapy for HBV-associated cirrhosis, the incidence of HBV-related VH and the associated mortality remains high (Kim et al., 2011). Therefore, it is necessary to develop a new prediction model to predict the risk of first VH occurrence in HBV-related cirrhotic patients with GEVs, especially in the context of widespread antiviral therapy.

Nomograms are visualization tools of prognosis evaluation, which can accurately and quantitatively predict the prognosis for individual patients (Iasonos et al., 2008; Balachandran et al., 2015). Currently, nomograms to predict clinical outcomes have been widely used in many medical fields to help in decision-making (Liang et al., 2015; Guo et al., 2022). However, to date, no ideal nomogram has been established to predict the risk of HBV-related VH. In the current study, our aim was to develop a nomogram specifically dedicated to predict the risk of first VH for HBV-related cirrhotic patients with GEVs and to validate its predictive performance in an independent group of patients.

## 2 Materials and methods

### 2.1 Ethical concerns

Approval was obtained from the Ethical Review Committee of the Beijing Ditan Hospital (Beijing, China). This study followed the ethical principles of the Declaration of Helsinki. Because this was a retrospective observational study, the Ethics Committee waived the need for informed consent. All sensitive patient information was anonymized and deidentified prior to analysis.

### 2.2 Study population

**Figure 1:**
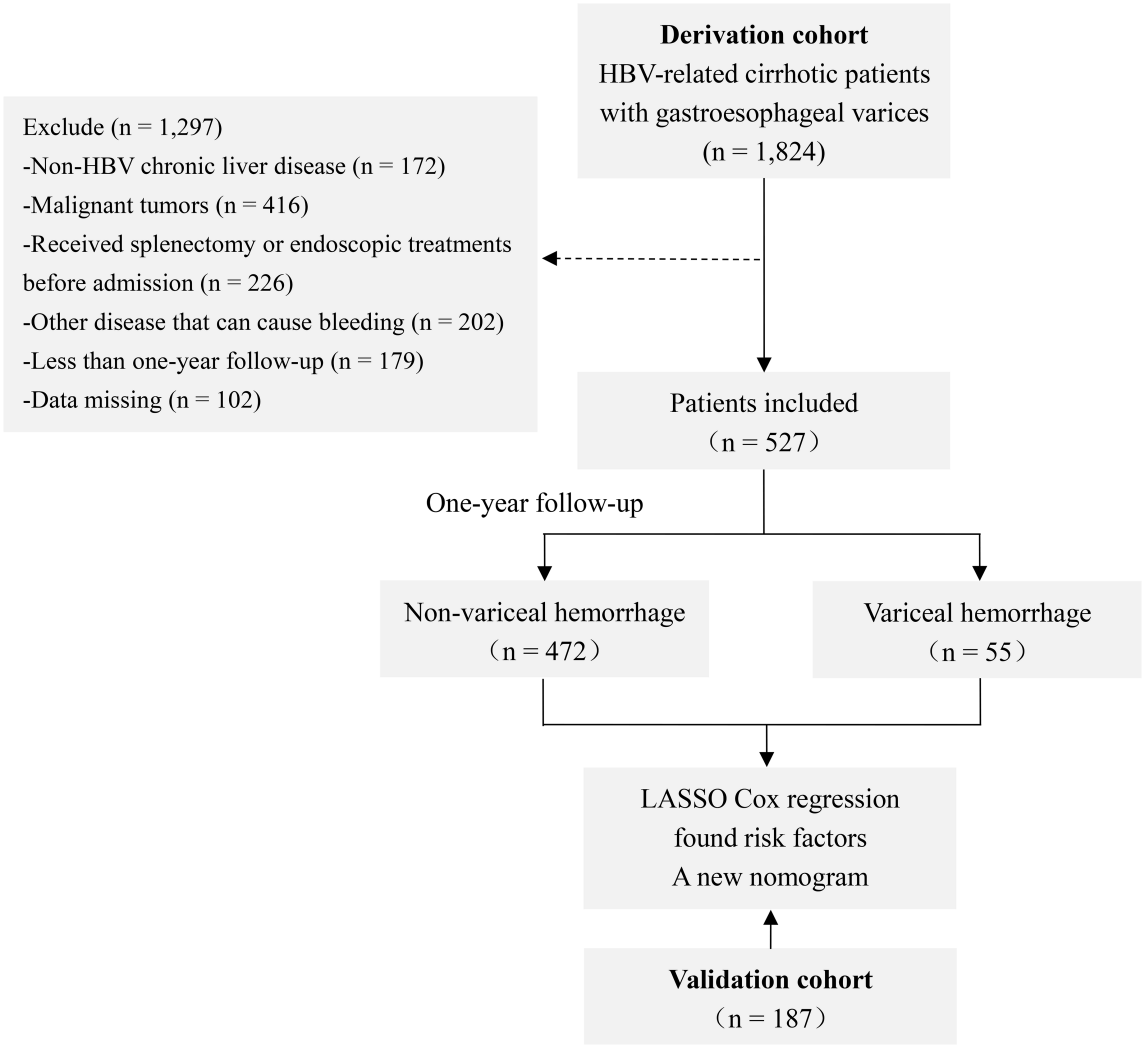
Study flow chart. Abbreviations: HBV, hepatitis B virus; LASSO, least absolute shrinkage and selection operator.

In total, data on 1,824 consecutive HBV-related cirrhotic patients with GEVs hospitalized at the Beijing Ditan Hospital of Capital Medical University from February 2008 to February 2021 were retrospectively screened. All subjects included in the study met the following inclusion criteria: (1) age at diagnosis ≥20 years; (2) serum hepatitis B surface antigen positive and under antiviral treatment for at least 6 months; (3) diagnosed with cirrhosis (based on clinical manifestations, imaging and blood tests, or liver biopsy); and (4) presence of GEVs confirmed through an endoscopic examination without previous history of VH. Patients with any of the following exclusion criteria were excluded from the study: (1) combined with other liver diseases (such as alcoholic hepatitis, other viral hepatitis, and autoimmune hepatitis); (2) complicated with liver cancer or other space-occupying lesions; (3) having undergone splenectomy, endoscopic treatments, or transjugular intrahepatic portosystemic shunts before inclusion in the study; (4) complicated with other conditions that may cause bleeding, such as ulcers and coagulation disorders; and (5) follow-up of less than one year or missing data. Eventually, 527 patients formed the derivation cohort. In addition, according to the same inclusion and exclusion criteria as the derivation cohort, 187 patients were selected from the Shuguang Hospital affiliated to the Shanghai University of Traditional Chinese Medicine between October 2015 and March 2019 to form a separate validation cohort (**Figure 1**).

### 2.3 Data collection

All study patients underwent detailed clinical, blood, endoscopic, and ultrasonographic evaluations within two days after admission, and relevant variables available were acquired from the electronic medical records. These variables included demographic characteristics (age and sex); complications (ascites, bacterial infection, and hepatic encephalopathy); routine laboratory parameters (aspartate aminotransferase, alanine aminotransferase, total bilirubin, γ-glutamyltransferase [GGT], alkaline phosphatase, albumin, white blood cell, red blood cell, platelet, neutrophil-lymphocyte ratio; hematocrit [HCT], potassium; sodium; blood urine nitrogen, creatinine; glucose, prothrombin time activity, prothrombin time, international normalized ratio, and HBV DNA levels), endoscopic parameters (the size of varices and RWM); and ultrasonography findings (portal vein diameter and spleen thickness). The Child-Pugh classification and model for end-stage liver disease (MELD) scores were calculated to evaluate the liver function status for each patient (Pugh et al., 1973; Kamath et al., 2001). All of the above variables were included in the least absolute shrinkage and selection operator (LASSO) Cox regression analysis to filter the candidate variables for the model. The NIEC and Rev-NIEC indexes were calculated according to previously published criteria (The North Italian Endoscopic Club for the Study and Treatment of Esophageal Varices, 1988; Merkel et al., 2000). All prognostic scores and definitions were applied at baseline.

### 2.4 Follow-up and outcome

The baseline date was defined as the date of admission to the hospital. All patients received regular outpatient or telephonic follow-ups and were hospitalized when needed. According to the guidelines, patients continued to receive antiviral treatment during the follow-up period, if indicated (Wang and Duan, 2021). All study subjects were followed up for one year (or until bleeding event occurred during the follow-up period). The end point of this study was the first gastric or esophageal VH occurrence confirmed by endoscopy. The VH diagnosis was based on the symptoms of hematemesis or/and melena with endoscopic variceal evidence or endoscopically evident active bleeding from gastric or esophageal varices without other possible source of hemorrhage (Garcia-Tsao et al., 2007).

### 2.5 Statistical analysis

Patient baseline characteristics were compared between the derivation and validation cohorts using the Mann-Whitney U test or Student’s *t-*test for continuous characteristics and the Pearson’s *χ2* test for categorical characteristics. Frequency (percentage) was reported to describe categorical characteristics, and median (interquartile ranges [IQR]) and mean ± standard deviation were reported to describe the continuous characteristics with skewed and normal distributions, respectively. Specific continuous variables were translated into categorized variables using the optimal cut-off value determined by the area under the receiver operating characteristic curve (AUC).

The LASSO regression analysis was performed to select the derivation cohort variables for inclusion in a multivariate Cox regression analysis to estimate the probability of VH (Tibshirani, 1997). The nomogram was elaborated according to the Cox regression coefficients of the identified prognostic factors. The discriminative capacity of the nomogram was assessed using AUC and concordance index (C-index) (Hanley and McNeil, 1982; Pencina and D’Agostino, 2015). The calibration curves were used to compare the consistency between actual observations and predicted probabilities (Van Calster et al., 2016; Alba et al., 2017). The decision curve analysis (DCA) curves were used to evaluate the clinical utility of the predictive model by quantifying the net benefit under different threshold probabilities (Vickers and Elkin, 2006). Patients were further classified into three risk groups using the 25th and 75th percentiles of the nomogram scores distribution as cut-off values (Papatheodoridis et al., 2020). Subsequently, we calculated the specificity, sensitivity, negative predictive value (NPV), and positive predictive value (PPV) for each cut-off. The cumulative VH incidence in the three risk groups was depicted using the Kaplan-Meier curves and compared using the modified log-rank test.

All statistical analyses were performed using the SPSS 22.0 statistical package (SPSS, Inc., Chicago, IL, USA) and R software (version 3.4.3). A two-tailed *P* value of <0.05 was considered to be statistically significant.

## 3 Results

### 3.1 Baseline characteristics of the study population

In total, 714 HBV-related GEV patients (527 *and 187* in the *derivation and validation cohorts, respectively*) were included in this study. A total of 55 (10.4%) and 20 (10.7%) patients in the derivation and validation cohorts, respectively, progressed to first VH occurrence at one-year follow-up. In the derivation cohort, the median age of patients was 55.0 years (IQR, 44.0–60.0 years) and 354 (67.2%) of them were males. The most common complication was ascites (57.3%), followed by bacterial infection (19.5%), and hepatic encephalopathy (3.8%). Endoscopy showed that the proportions of small, medium, and large varices were 44.4%, 27.5%, and 28.1%, respectively. RWM was observed in 34.9% of the patients. Most of the patients belonged to the Child-Pugh grade B (50.3%), followed by grade A (32.3%), and grade C (17.5%), while the median MELD score was 10.0 (IQR, 8.0–13.0). While comparing these baseline characteristics of the derivation cohort with that of the validation cohort, we found that patients in the derivation cohort had higher rates of bacterial infection and large varices (*P <*0.05) and had lower international normalized ratio level (*P <*0.05). Further details on clinical features of the patients in the two cohorts are summarized in **Table 1**.

**Table:** Table 1 *Baseline characteristics of the patients in the derivation and validation cohorts*.

Variables	Derivation cohort	Validation cohort	*P* value
	(n = 527)	(n = 187)	
Age (years)	55.0 (44.0-60.0)	52.0 (43.0-58.0)	0.202
Male sex	354 (67.2)	135 (72.2)	0.204
Ascites	302 (57.3)	106 (56.7)	0.883
Hepatic encephalopathy	20 (3.8)	7 (3.7)	0.975
Bacterial infection	103 (19.5)	20 (10.7)	0.006
The size of varices			0.027
Small	234 (44.4)	92 (49.2)	
Medium	145 (27.5)	61 (32.6)	
Large	148 (28.1)	34 (18.2)	
Red wale marks	184 (34.9)	63 (33.7)	0.762
Alanine aminotransferase (U/L)	36.8 (24.5-64.4)	35.4 (24.4-78.5)	0.702
Aspartate aminotransferase (U/L)	48.6 (33.2-87.5)	47.9 (32.8-91.8)	0.900
total bilirubin (µmol/L)	29.6 (18.6-47.4)	28.9 (17.4-57.4)	0.785
γ-Glutamyltransferase (U/L)	53.2 (28.6-110.7)	50.6 (26.1-128.5)	0.676
alkaline phosphatase (U/L)	99.3 (73.1-135.5)	98.2 (77.7-133.2)	0.835
Albumin (g/L)	31.4 (27.7-36.6)	31.1 (27.1-36.4)	0.593
White blood cell (×10^9^/L)	3.3 (2.5-4.7)	3.3 (2.5-4.5)	0.678
Red blood cell (×10^12^/L)	3.2 (3.7-4.1)	3.2 (3.6-4.2)	0.584
Platelet (×10^9^/L)	59.9 (43.6-85.0)	60.3 (44.7-84.0)	0.712
Neutrophil-lymphocyte ratio	1.9 (1.4-2.9)	1.9 (1.3-3.1)	0.988
Hematocrit (%)	35.0 (31.2-38.7)	34.8 (30.6-39.6)	0.965
Potassium (mmol/L)	3.7 (3.5-4.0)	3.7 (3.4-4.0)	0.595
Sodium (mmol/L)	140.1 (137.8-141.8)	140.0 (137.9-142.0)	0.841
Blood urine nitrogen (mmol/L)	5.0 (4.0-6.2)	5.0 (4.1-6.4)	0.502
Creatinine (µmol/L)	63.0 (55.0-74.7)	63.0 (55.0-73.0)	0.689
Glucose (mmol/L)	5.6 (5.0-7.0)	5.6 (4.9-7.5)	0.858
Prothrombin time (s)	14.6 (13.1-16.3)	15.1 (13.4-16.9)	0.074
Prothrombin time activity (%)	66.6 (55.0-78.0)	64.0 (52.8-75.1)	0.099
International normalized ratio	1.2 (1.1-1.4)	1.3 (1.1-1.5)	0.003
HBV DNA (log_10_IU/ml)	0.0 (0.0-3.6)	0.0 (0.0-3.9)	0.737
Spleen thickness (mm)	48.0 (42.0-56.0)	49.0 (42.0-55.0)	0.726
Portal vein diameter (mm)	12.0 (11.0-13.0)	12.0 (11.0-13.0)	0.405
Child-Pugh grade			0.299
A	170 (32.3)	54 (28.9)	
B	265 (50.3)	91 (48.7)	
C	92 (17.5)	42 (22.5)	
MELD score	10.0 (8.0-13.0)	10.2 (8.0-14.0)	0.424

Data are presented as n (%) or median (interquartile range).

MELD, model for end-stage liver disease.

### 3.2 Independent prognostic factors for VH

**Figure 2:**
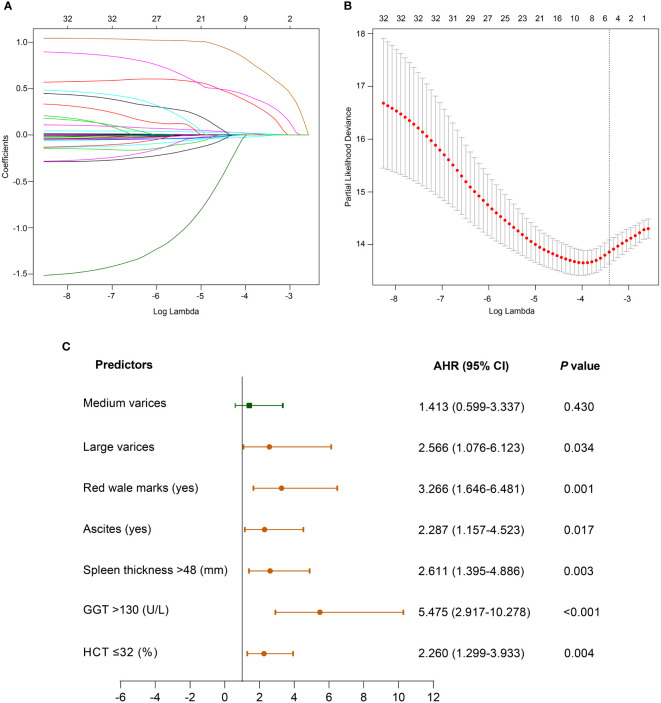
Clinical characteristics selection using the LASSO Cox regression model. **(A)** LASSO coefficient profiles of the 32 variables. **(B)** 10-fold cross-validation is applied to select the most suitable parameter using LASSO regression model. The vertical line on the right is plotted with six selected variables, which constructed a more concise model within one standard error. **(C)** Forest plot of multivariate Cox regression analysis of the selected variables in the derivation cohort. Abbreviations: AHR, adjusted hazard ratio; CI, confidence interval; GGT, γ-glutamyltransferase; HCT, hematocrit; LASSO, least absolute shrinkage and selection operator.

The LASSO regression output showed that the size of varices, RWM, ascites, spleen thickness, GGT, and HCT obtained from the derivation cohort were significant predictors of the first VH when the lambda was one standard error (**Figures 2A, B**). To elucidate the relationship between these potential predictors and the VH outcome, we further performed a multivariable Cox regression analysis using these six factors. However, the results revealed that the adjusted hazard ratios (AHR) of spleen thickness, GGT, and HCT were close to 1 (**Supplementary Figure 1**); thus, these three continuous variables were converted into categorical variables according to the optimal cut-off value. After adjustment, the above six categorical variables were reincorporated into the Cox regression analyses. Finally, we screened six independent categorical predictors in the derivation cohort: large varices (AHR, 2.566; 95% confidence interval [CI], 1.076–6.123; *P* = 0.034), RWM (AHR, 3.266; 95% CI, 1.646–6.481; *P* = 0.001), ascites (AHR, 2.287; 95% CI, 1.157–4.523; *P* = 0.017), spleen thickness >48 mm (AHR, 2.611; 95% CI, 1.395–4.886; *P* = 0.003), GGT >130 U/L (AHR, 5.475; 95% CI, 2.917–10.278; *P <*0.001), and HCT ≤32% (AHR, 2.260; 95% CI, 1.299–3.933; *P* = 0.004) (**Figure 2C**).

### 3.3 Construction and validation of the prognostic nomogram for the VH prediction

**Figure 3:**
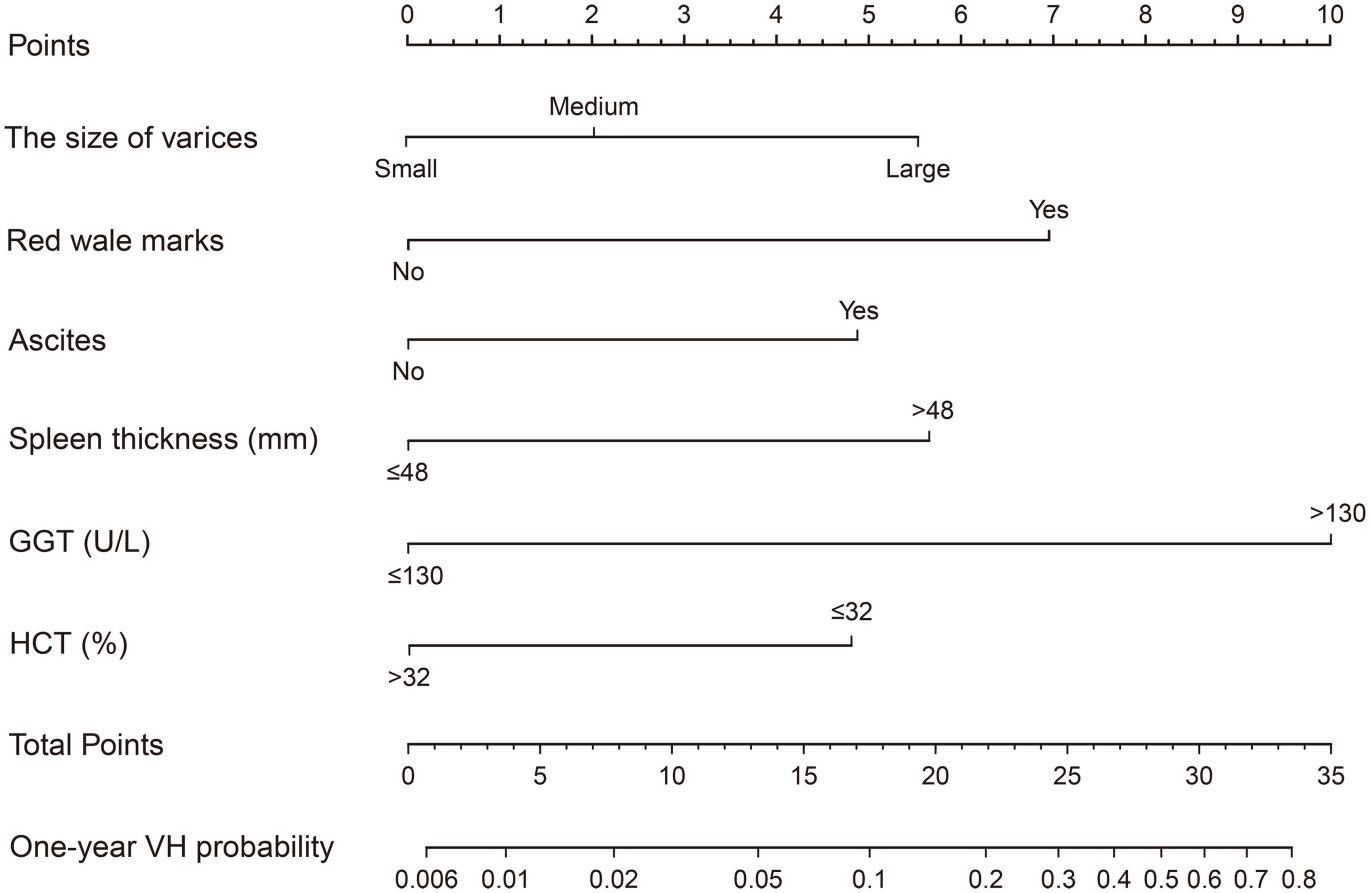
Nomogram for predicting the probability of VH within one-year in HBV-related cirrhotic patients with GEVs. The total points are calculated by adding the points of each covariate corresponding to the point on the upper pointing axis. A line is then drawn down to the probability axis at the bottom of the nomogram, which indicated the VH probability within one-year. Abbreviations: GGT, γ-glutamyltransferase; HCT, hematocrit; VH, variceal hemorrhage; HBV, hepatitis B virus; GEV, gastroesophageal varice.

**Figure 4:**
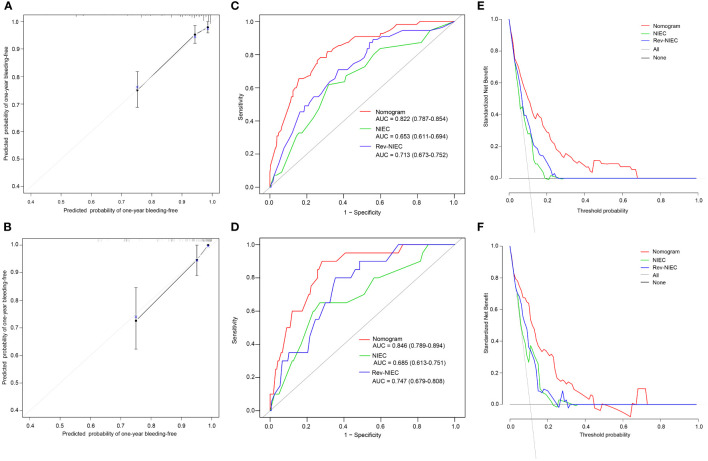
Calibration plots, ROC curves, and DCA curves for predicting VH in HBV-related cirrhotic patients with GEVs. The calibration plots of the nomogram for VH in the derivation **(A)** and validation cohorts **(B)**, in which the predicted probability of VH was compared with the actual bleeding probability. The ROC curves of the nomogram, NIEC index, and Rev-NIEC index in the derivation **(C)** and validation cohorts **(D)**. The DCA curves in the derivation **(E)** and validation cohorts **(F)** show that the net benefit of the nomogram is greater than the net benefit of the NIEC and Rev-NIEC indexes. Abbreviations: AUC, areas under the receiver operating characteristic curve; NIEC, North Italian Endoscopic index; Rev-NIEC, revised NIEC index; ROC, receiver operating characteristic; DCA, decision curve analysis; VH, variceal hemorrhage; HBV, hepatitis B virus; GEV, gastroesophageal varice.

Based on the influential factors selected from the derivation cohort, a nomogram was developed to predict the risk of first VH occurrence within one year in HBV-related cirrhotic patients with GEVs (**Figure 3**). The detailed score for each variable and the estimated risk of first VH occurrence according to the nomogram score in the derivation cohort are presented in **Supplementary Figure 2**. The application of the nomogram is as follows: each prediction indicator was assigned a corresponding score based on its value on the nomogram. After calculating the total score, we draw a vertical line using the total score and the predicted risk corresponding to the total score is the individual probability of variceal bleeding. For example, one patient had larger varices (5.6 points) and RWM (6.9 points) diagnosed at endoscopy; ultrasonography examination revealed ascites (4.9 points) and spleen thickness was 35mm (0 points); blood tests showed GGT was 200U/L (10 piont) and HCT was 35% (0 points). His total points was 5.6 + 6.9+4.9+0+10+0 = 27.4, and the corresponding probability of bleeding within 1-year was 0.44 (44%) (**Supplementary Figure 3**). The C-index of the nomogram were 0.806 (95% CI, 0.753–0.859) and 0.820 (95% CI, 0.742–0.898) in the derivation and validation cohorts, respectively, which suggested a good discrimination potential of this nomogram. We also created a calibration plot, which indicated high consistency between the predicted outcomes and actual observations in the derivation (**Figure 4A**) and external validation cohorts (**Figure 4B**).

### 3.4 Comparison of the prognostic nomogram with NIEC and Rev-NIEC indexes

To compare the discriminative ability of the constructed nomogram with those of the traditional (NIEC and Rev-NIEC) indexes, we compared the AUC values of each model. The nomogram had an AUC value of 0.822 (95% CI, 0.787–0.854), which was significantly higher than that of the NIEC index (AUC, 0.653; 95% CI, 0.611–0.694) and Rev-NIEC index (AUC, 0.713; 95% CI, 0.673–0.752) in the derivation cohort (*P <*0.05 for each model) (**Figure 4C**). Moreover, the nomogram had the highest AUC value of 0.846 (95% CI, 0.786–0.894) compared with that of the NIEC index (0.685; 95% CI, 0.613–0.751) and Rev-NIEC index (0.747; 95% CI, 0.679–0.808) in the validation cohort (*P <*0.05 for both) (**Figure 4D**). We also used DCA to compare the net clinical benefit of these models. As shown in **Figures 4E, F**, compared with the NIEC and Rev-NIEC indexes, the new nomogram provided greater net benefits both in the derivation and validation cohorts.

### 3.5 Performance of the nomogram in stratifying risk of patients

**Figure 5:**
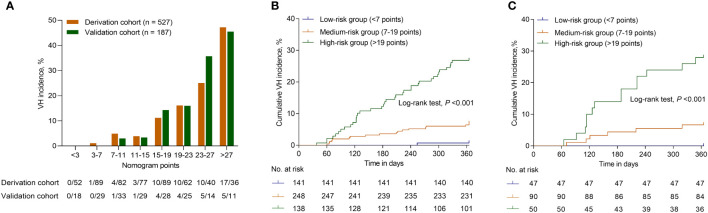
Risk for VH of patients with HBV-related GEVs with different scores. **(A)** The bars show the VH incidence for different score category in the derivation and validation cohorts. The cumulative VH incidence for patients categorized into different risk groups according to the nomogram in the derivation **(B)** and validation cohorts **(C)**. Abbreviations: VH, variceal hemorrhage; HBV, hepatitis B virus; GEV, gastroesophageal varice.

As the nomogram points increased, the VH incidence also increased (**Figure 5A**). For convenience in the clinical evaluation, based on the 25th and 75th percentiles of the score distribution, *i.e.* <7, 7–19, and >19 points, 141 (26.8%), 248 (47.1%), and 138 (26.2%) patients were stratified into low-, medium-, and high-risk groups, respectively, in the derivation cohort. Moreover, in the derivation cohort, compared with patients in the low-risk group, those in the medium-risk and high-risk groups were at a significantly higher risk of VH, with a hazard ratio (HR) of 10.022 (95% CI, 1.334–75.306; *P* = 0.025) and 43.518 (95% CI, 5.970–317.217; *P <*0.001), respectively. In the derivation cohort, the cumulative VH incidence was 0.7%, 6.9%, and 26.8% in the low-, medium-, and high-risk groups, respectively (log-rank test, *P <*0.001; **Figure 5B**); and in the validation cohort, the cumulative incidence was 0.0%, 6.7%, and 28.0%, respectively (log-rank test, *P <*0.001; **Figure 5C**). The low cut-off value (>7) of the nomogram score achieved 98.2% sensitivity (95% CI, 90.3%–100.0%) and 99.3% NPV (95% CI, 96.3%–100.0%) in the derivation cohort and offered 100% sensitivity and NPV in the validation cohort to exclude patients who did not develop VH within one year (**Table 2**).

**Table 2:** Sensitivity, specificity, and predictive values of the nomogram classification determined by the 25th and 75th percentiles of the score distribution.

Cut-off value	Sensitivity (%) (95% CI)	Specificity (%) (95% CI)	PPV (%) (95% CI)	NPV (%) (95% CI)
Derivation cohort (n = 527)				
Nomogram <7	98.2 (90.3-100.0)	31.4 (27.2-35.8)	14.3 (10.9-18.2)	99.3 (96.3-100.0)
Nomogram <19	67.3 (53.3-79.3)	78.8 (74.8-82.4)	27.0 (19.8-35.3)	95.4 (92.8-97.2)
Validation cohort (n = 187)				
Nomogram <7	100.0 (83.2-100.0)	28.1 (21.5-35.6)	14.3 (8.9-21.2)	100.0 (92.5-100.0)
Nomogram <19	70.0 (45.7-88.1)	78.4 (71.4-84.4)	14.0 (8.2-21.8)	95.6 (90.7-98.4)

The patients were stratified into three groups according to the 25th percentile and the 75th percentile of the nomogram-predicted score: low-risk group (<25th percentile), medium-risk group (25th–75th percentile), and high-risk group (>75th percentile). The 25th percentile was 7 and the 75th percentile 19 points.

CI, confidence interval; PPV, positive predictive value; NPV, negative predictive value.

## 4 Discussion

In the current study, we developed and validated an individualized nomogram model specifically for HBV-related cirrhotic patients with GEVs to assess the risk of first VH occurrence within one-year. The robustness and predictive power of this nomogram was demonstrated to be superior compared with the traditional NIEC and Rev-NIEC indexes by internal and external validation tests. Importantly, this nomogram could be used as a risk stratification tool to identify patients at different risk levels of VH occurrence, which helps to promote the precise prevention of VH occurrence and personalized management of patients with GEVs.

VH prevention is the primary goal in the GEV patient management (Garcia-Tsao et al., 2017; de Franchis et al., 2022). Several decades ago, researchers noticed the importance of bleeding risk assessment in the development of prevention strategies and have established several models for this purpose (The North Italian Endoscopic Club for the Study and Treatment of Esophageal Varices, 1988; Merkel et al., 2000; Tacke et al., 2007). Among them, the NIEC index is the most widely used, and the risk stratification based on this index is still an important basis for primary prevention strategies (The North Italian Endoscopic Club for the Study and Treatment of Esophageal Varices, 1988; Garcia-Tsao et al., 2017; de Franchis et al., 2022). However, the predictive power of such models are expected to vary among different populations owing to differences in the etiologies of cirrhosis. Based on our research, the predictive performance of the traditional NIEC and Rev-NIEC indexes for patients with HBV-related GEVs is lower than that for the patients with hepatitis C virus/alcohol abuse-related GEVs studied in the original report (The North Italian Endoscopic Club for the Study and Treatment of Esophageal Varices, 1988; Merkel et al., 2000). Furthermore, it is reported that the etiology is an independent VH predictor (Tacke et al., 2007), indicating different progression of VH between different etiologies. Therefore, different evaluation model based on different etiologies may be more effective for VH risk prediction. For patients with HBV infection, antiviral therapy is a very important strategy regardless of the disease stage, which can delay disease progression and improve long-term prognosis (Iloeje et al., 2006; Chen et al., 2006; European Association for the Study of the Liver, 2017; Wang and Duan, 2021). Previous studies have also demonstrated that antiviral therapy is effective in reducing the VH rate (Li et al., 2013; He et al., 2019). Nevertheless, the incidence and mortality of HBV-related VH remains high (Kim et al., 2011). Thus, apart from the antiviral therapy, it is essential to develop other targeted prevention strategies in accordance with bleeding risk of each individual, for reducing the HBV-associated first VH occurrence.

In the current study, we carefully analyzed data from 527 HBV-related cirrhotic patients with GEVs and explored the VH-related impact factors based on one-year follow-up outcomes. As expected from previous literature (The North Italian Endoscopic Club for the Study and Treatment of Esophageal Varices, 1988; Kleber et al., 1991; Merkel et al., 2000; Kim et al., 2019), the sizes of varices as well as the presence of RWM were strong predictors for VH. Endoscopy, as the gold standard technique for the diagnosis of varices, plays an important role in the bleeding risk assessment for patients with GEVs (de Franchis et al., 2022). Patients with large varices have approximately three times higher risk of VH than those with small varices, while the presence of RWM increased the risk of bleeding up to four times (Park et al., 2004; Aggeletopoulou et al., 2018). Further, the liver dysfunction severity, e.g., elevated GGT levels and ascites, was found to be an essential risk factor for VH. However, the liver function indicators were different from the previous studies (The North Italian Endoscopic Club for the Study and Treatment of Esophageal Varices, 1988; Merkel et al., 2000), in which the Child-Pugh classification as a liver function indicator was associated with bleeding. This may be because the Child-Pugh scoring system involves some subjective indexes (hepatic encephalopathy and ascites) and interrelated indexes (serum albumin and ascites) (Pugh et al., 1973), which virtually increase the instability of the prediction in different studies. It has also been previously reported that the Child-Pugh classification is not associated with the bleeding incidence (Kleber et al., 1991). Besides such well-recognized factors mentioned above, HCT and splenic thickness were also found as important factors affecting VH occurrence. HCT is one of the most important indicators of whole blood viscosity (Voerman and Groeneveld, 1989). A decreased blood viscosity is associated with higher bleeding risk and increased bleeding severity (Ohki et al., 1988). Previous reports also indicated that low hematocrit is a risk factor for variceal bleeding (Liu et al., 2006; Zhou et al., 2017). Splenomegaly, excluding diseases of the blood system, is an important manifestation of portal hypertension in cirrhosis (Li et al., 2017). The increased spleen thickness indicates the severity of liver morphological changes, indicating the portal hypertension and gastroesophageal variceal bleeding progression (Bolognesi et al., 2002; Berzigotti et al., 2008; Zhang et al., 2019).

We combined the above mentioned six independent predictors and established a novel and easy-to-use nomogram to evaluate the probability of the first VH occurrence. To our knowledge, this is the first report on developing a nomogram for predicting the risk of VH in HBV-related cirrhotic patients with GEVs. This established nomogram demonstrated better predictive performance for VH occurrence compared with the traditional NIEC and Rev-NIEC indexes, as supported by the AUC and DCA curves in the internal and external validation tests. Thus, this nomogram developed specifically to report that GEVs screening strategies for HBV-related cirrhosis offers unique advantages over the traditional risk predicting models.

For efficient prophylaxis, accurate risk stratification and prognosis assessment are important because patients in the high-risk group may benefit from earlier prevention strategies, while patients in the low-risk group could avoid undergoing unnecessary interventions (Tacke et al., 2007). In our study, we stratified patients into low- (<7 points), medium- (7–19 points), and high- (>19 points) risk groups according to their bleeding risk scores. In this hierarchical model, 26.8% of patients were categorized as low-risk group with an annual bleeding risk of 0.7%. Furthermore, the low cut-off point of 7 offered 98.2% sensitivity and 99.3% NPV for VH prediction in the derivation cohort, and 100% sensitivity and NPV in the validation cohort, which means that endoscopic screening and preventive measure should be reduced in patients with low-risk group features to avoid the risk of overdiagnosis and overtreatment. In contrast, the 26.8% annual VH incidence in the high-risk group signifies that patients in this group require closer surveillance and proactive preventive strategies, and it can be accepted that the higher the risk score, the more attention should be given. Therefore, the clinical significance of this study lies in the idea that clinicians can use this nomogram for assistance in understanding the risk for VH and choosing appropriate and individualized treatment strategies according to patient risk level, instead of adhering to a single treatment regimen, which may contribute to improved overall outcomes in all patients with GEVs and allocate medical resources efficiently.

This study has several limitations. First, this is a retrospective study and potential selection bias is inevitable. However, the nomogram was derived from a large sample size cohort of tertiary hospital and it was validated in an external cohort. Nonetheless, data from multicenter prospective studies with larger sample size are still needed to make the results generalizable. Second, the occurrence of bleeding events may be influenced by precipitating factors, such as severe coughing, and constipation. However, this challenge reflects those faced in daily clinical practice and also exists in all large-scale research studies. Although these precipitating factors may shorten the period of the VH event, this shortened period was considered meaningful for early warning of bleeding. Finally, the follow-up duration was one-year, and the model’s predictive performance for the long-term prognosis remains unclear. Conversely, one-year is a reasonable time span for the development of VH risk prediction model. Despite these limitations, our research provides new guidance for the selection of prevention strategies for HBV-related VH and offers an idea to develop a predictive model for VH in patients with GEVs from other etiologies.

## 5 Conclusions

In summary, the nomogram established in our study can be useful for estimating the first VH probability within one-year and stratifying the bleeding risks in HBV-related cirrhotic patients with GEVs, which can assist clinicians in determining the appropriate prophylactic strategies. However, the clinical utility and true predictive value of this nomogram needs to be further verified in larger prospective studies.

## Data availability statement

The raw data supporting the conclusions of this article will be made available by the authors, without undue reservation.

## Ethics statement

The studies involving human participants were reviewed and approved by Ethical Review Committee of the Beijing Ditan Hospital (Beijing, China). Written informed consent for participation was not required for this study in accordance with the national legislation and the institutional requirements. Written informed consent was not obtained from the individual(s) for the publication of any potentially identifiable images or data included in this article.

## Author contributions

XW, YM, and QZ conceived and designed the project. QZ, SN, KS, XZ, YZ, and YB collected the data. QZ, BZ, and LY analyzed and interpreted the data. QZ, SN, and LY drafted the manuscript. All authors contributed to the article and approved the submitted version.
