# Supplementary material for: A novel prognostic model for predicting the risk of first variceal hemorrhage in patients with HBV-related cirrhosis
Source: Front Cell Infect Microbiol. 2023 Jan 17;13:1062172. doi: 10.3389/fcimb.2023.1062172 (PMC9886685; doi:10.3389/fcimb.2023.1062172)
Supplement: Supplementary file 1 [file DataSheet_1.docx]

Supplementary Material

# Supplementary Figures and Tables

## Supplementary Figures


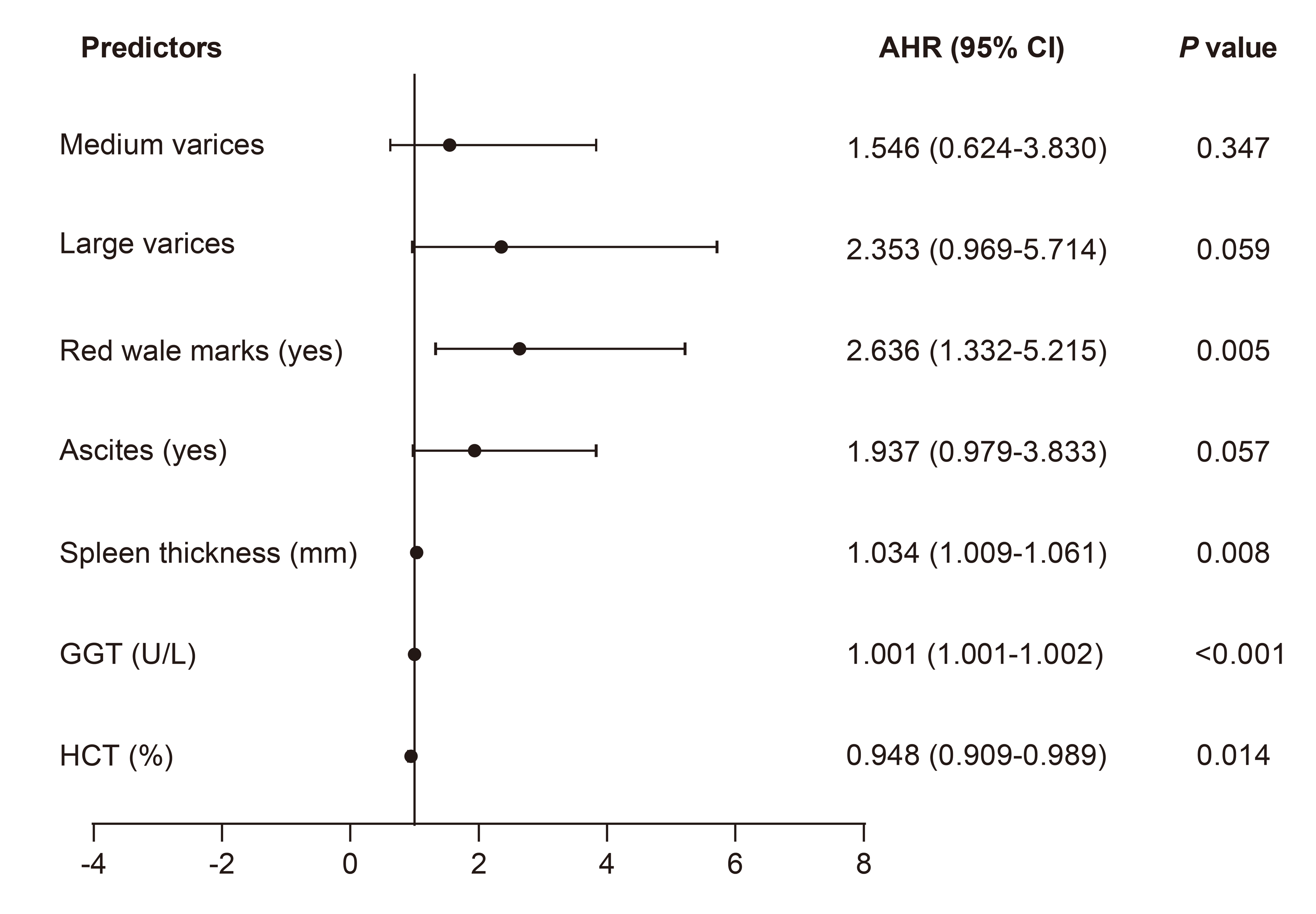


**Supplementary Figure 1.** The forest plot of multivariate Cox regression analysis of selected variables in the derivation cohort (where GGT, HCT, and spleen thickness are continuous variables). Abbreviations: AHR, adjusted hazard ratio; CI, confidence interval; GGT, γ-glutamyltransferase; HCT, hematocrit.


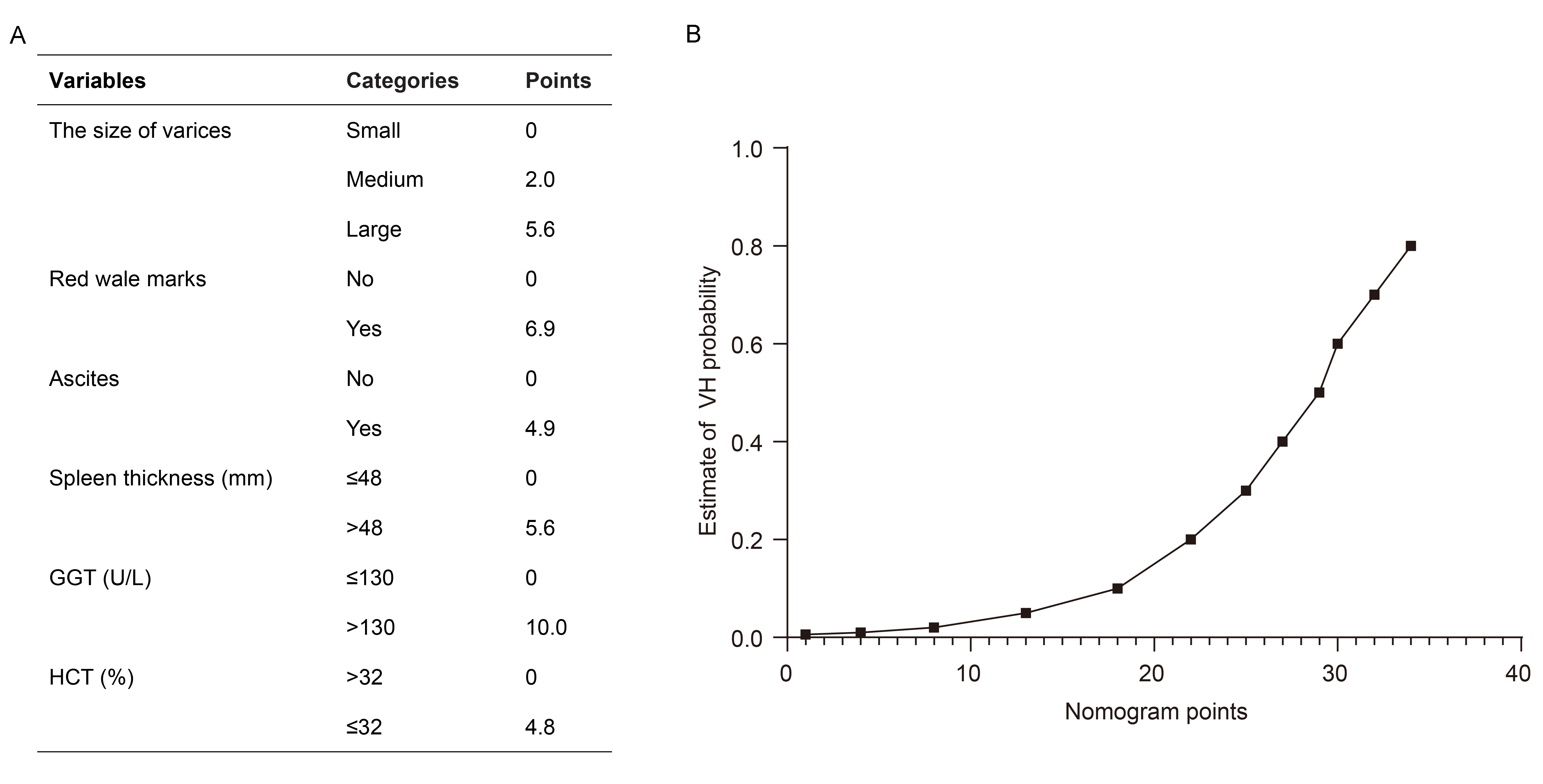


**Supplementary Figure 2.** Rating form of the nomogram (A) and estimated probability of VH in the derivation cohort (B). Abbreviations: GGT, γ-glutamyltransferase; HCT, hematocrit; VH, variceal hemorrhage.


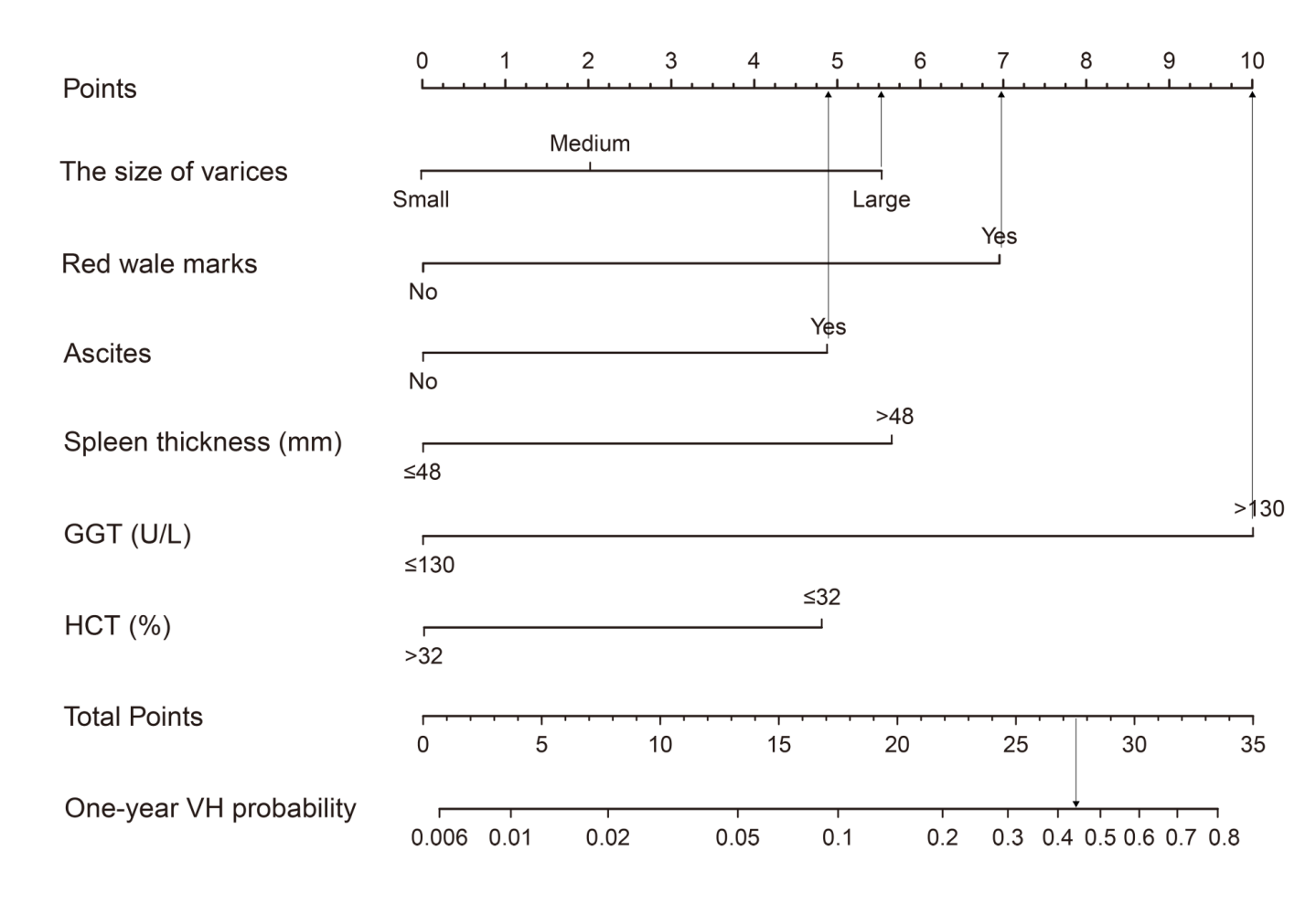


**Supplementary Figure 3**. Example prediction nomogram for risk of variceal bleeding in HBV-related cirrhotic patients with GEVs. Abbreviations: GGT, γ-glutamyltransferase; HCT, hematocrit; VH, variceal hemorrhage.
